# Dimer Interface in Natural Variant NK1 Is Dispensable for HGF-Dependent Met Receptor Activation

**DOI:** 10.3390/ijms22179240

**Published:** 2021-08-26

**Authors:** Yumiko Tahira, Katsuya Sakai, Hiroki Sato, Ryu Imamura, Kunio Matsumoto

**Affiliations:** 1 Division of Tumor Dynamics and Regulation, Cancer Research Institute, Kanazawa University, Kakuma, Kanazawa 920-1192, Japan; yu23@stu.kanazawa-u.ac.jp (Y.T.); hiroki.sato@staff.kanazawa-u.ac.jp (H.S.); imamura@staff.kanazawa-u.ac.jp (R.I.); 2 WPI-Nano Life Science Institute (WPI-NanoLSI), Kanazawa University, Kakuma, Kanazawa 920-1192, Japan; 3 Institute for Frontier Science Initiative, Kanazawa University, Kakuma, Kanazawa 920-1192, Japan

**Keywords:** growth factor, HGF, Met, receptor tyrosine kinase

## Abstract

NK1, a splicing variant of hepatocyte growth factor (HGF), binds to and activates Met receptor by forming an NK1 dimer and 2:2 complex with Met. Although the structural mechanism underlying Met activation by HGF remains incompletely resolved, it has been proposed that the NK1 dimer structure participates in this activation. We investigated the NK1 dimer interface’s role in Met activation by HGF. Because N127, V140, and K144 are closely involved in the head-to-tail NK1 dimer formation, mutant NK1 proteins with replacement of these residues by alanine were prepared. In Met tyrosine phosphorylation assays, N127-NK1, V140-NK1, and K144-NK1 showed 8.3%, 23.8%, and 52.2% activity, respectively, compared with wild-type NK1. Although wild-type NK1 promoted cell migration and scattering, N127-NK1, V140-NK1, and K144-NK1 hardly or marginally promoted them, indicating loss of activity of these mutant NK1 proteins to activate Met. In contrast, mutant HGFs (N127-HGF, V140-HGF, and K144-HGF) with the same amino acid replacements as in NK1 induced Met tyrosine phosphorylation and biological responses at levels comparable to those of wild-type HGF. These results indicate that the structural basis responsible for NK1-dependent Met dimer formation and activation differs from, or is at least distinguishable from, the structural basis responsible for HGF-dependent Met activation.

## 1. Introduction

Hepatocyte growth factor (HGF) and its receptor, the Met receptor tyrosine kinase, play important roles in embryonic development, tissue regeneration, and tumor progression [1,2,3]. Based on these physiological and pathogenic functions, HGF-Met signaling has been a target in drug discovery for both regeneration-based therapeutics and anticancer therapeutics [1,2,3]. Clinical trials of recombinant HGF for the treatment of patients with fulminant hepatitis, spinal cord injury, and amyotrophic lateral sclerosis have been completed or are ongoing [4]. HGF gene therapy was approved for the treatment of patients with critical limb ischemia [5]. The Met tyrosine kinase inhibitors capmatinib and tepotinib were approved in 2020 as anticancer drugs for the treatment of non-small cell lung cancer patients with Met exon 14 skipping [6,7]. In combination with new drug modalities and technologies, structural elucidation of the mechanism by which HGF activates Met has led to the discovery of Met-agonistic and -inhibitory drugs with superior ability and/or different chemical characteristics compared with recombinant HGF protein and small inhibitory molecules [6,7,8,9,10,11]. However, an understanding of the structural basis explaining how HGF activates Met has remained elusive.

**Figure 1:**
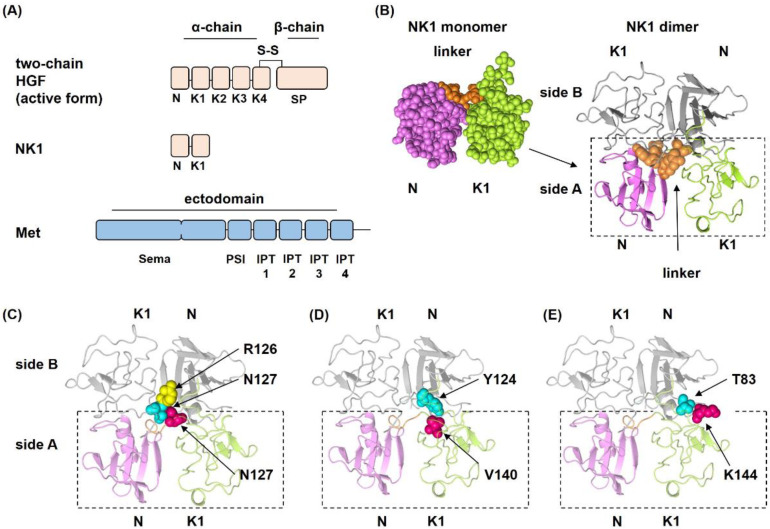
Domain structures of HGF, NK1, and the ectodomain of Met (**A**), NK1 dimer structure (**B**), and positions of amino acids at which replacement mutations were introduced (**C**–**E**). In A, HGF is a disulfide-linked heterodimer composed of an α-chain and a β-chain. Met has six domains in its ectodomain: the large N-terminal region is the Sema domain, followed by the PSI and IPT1-4 domains. In B, the N domain is shown in purple, the K1 domain in green, and the linker region in orange. The left is a space-filling model of the NK1 monomer. NK1 has a short linker consisting of K122 to N127 that connects the N and K1 domains. The NK1 dimer is shown on the right in a ribbon model, in which side A represents the same region as the space-filling model. In B–E, an individual NK1 is shown in color or gray. In C–E, amino acid residues replaced with Ala are shown in magenta. N127 forms two hydrogen bonds with R126 (yellow) and N127 (blue) in another NK1. V140 in the K1 domain forms a hydrophobic bond with Y124 (blue) in another NK1, and K144 forms a hydrogen bond with T83 (blue) in another NK1. Structures were prepared using PDB 1NK1.

HGF is a disulfide-linked protein composed of an α-chain, which is composed of an N-terminal (N) and four kringle domains (K1–K4), and a β-chain, also known as the serine protease-like (SP) domain (Figure 1A) [12,13]. Biochemical and crystallographic approaches revealed that HGF has two individual binding regions for Met, the NK1 domain and the SP domain [14,15,16,17]. While the binding interface between the SP domain and Sema domain of Met (Figure 1A) has been revealed by crystallographic analysis [17], the crystal structure of the complex between NK1 and Met has not been obtained, despite the binding of NK1 to Met [15,16,18]. NK1 is one of the splicing variants of HGF, and notably, NK1 harbors agonist activity for Met [15,16,19,20]. Indeed, crystal structures of NK1 revealed a head-to-tail dimer configuration, and, importantly, this NK1 dimer interface is required for the agonist activity of NK1 [15,16]. NK1 associates with the Sema-PSI domains of Met (Figure 1A) [15,18], and analysis of the complex of NK1 and Sema-PSI domains by small-angle X-ray scattering (SAXS) has indicated a tetramer complex with 2:2 stoichiometry [15]. Recent analysis by cryo-electron microscopy (cryo-EM) also showed that NK1 forms a head-to-tail dimer and recruits two c-Met molecules symmetrically to both sides by the interaction between the K1 domain and the Sema domain of Met [21].

A structural model of Met activation by HGF has been proposed in which HGF binds to Sema-PSI domains in Met through both the NK1 domain [15,16] and the SP domain [17], forms an HGF dimer through a head-to-tail dimer of the NK1 region, and leads to a 2:2 complex of HGF and Met [22]. The 2:2 complex composed of HGF and Sema-PSI domains was proposed by SAXS analysis, in which the NK1 dimer structure was applied as a best-fitted model [22]. However, HGF was shown to form a complex with the entire ectodomain of Met with 1:1 stoichiometry in electron microscopic analysis, SAXS analysis [22], and size-exclusion chromatography [23]. No crystal structure for the 2:2 complex of the HGF and Met ectodomain has been reported. Furthermore, in contrast to what had been believed for years, we reported that the Met-binding site in the K1 domain is dispensable for Met activation by HGF, while the presence of the NK1 region is still required for Met activation [23]. The analysis based on the recent cryo-EM data showed that HGF binds to Met in a 1:2 ratio [21]. This complexity explains why an understanding of how HGF activates Met is still elusive, and the role of the NK1 domain in HGF-dependent Met activation remains unclear. In this report, we address the involvement of the dimer configuration in Met activation by HGF and NK1. The results indicate that NK1 dimer formation is indispensable for NK1-dependent Met activation, but unexpectedly it is dispensable in HGF-dependent Met activation.

## 2. Results

### 2.1. Preparation of Wild-Type and Mutant HGF and NK1 Proteins

Crystal structures of the NK1 dimer revealed amino acid residues that are strongly involved in NK1 dimer formation (Figure 1) [24]. Several noncovalent bonds are formed between each NK1 molecule in a complementary manner in the dimer interfaces, by which a particular head-to-tail NK1 dimer is spatially arranged. Based on these structures, the individual amino acids that participate in NK1 dimer formation, N127, V140, and K144, were replaced with Ala (Figure 1C–E). N127 is the amino acid in the linker sequence that connects the N and K domains (Figure 1B). N127 is located at the center of the dimer interface and connects with a second NK1 by two hydrogen bonds with R126 and N127 in the second NK1 (Figure 1C) [24]. Amino acid replacement experiments indicated that N127 is important for the agonistic activity of NK1 [25]. V140 in the K1 domain participates in a hydrophobic bond with Y124 in another NK1 (Figure 1D) [24]. K144 in the K1 domain forms a hydrogen bond with T83 in the N domain of another NK1 (Figure 1E) [24].

Wild-type HGF and NK1 and the HGF and NK1 mutants were expressed in human cells; the recombinant proteins were purified by heparin-affinity chromatography and subsequent cation exchange chromatography (Figures S1 and S2). Protein staining and Western blotting indicated that wild-type and mutant HGF and NK1 proteins were prepared to levels suitable for biological assays (Figure S3). Both wild-type and mutant HGF showed a single band in non-reducing conditions and two bands in reducing conditions, as expected (Figure S3B). Because HGF is glycosylated, recombinant HGFs showed some heterogeneity in SDS-PAGE; however, the glycosylation does not affect the biological activities of HGF [26].

### 2.2. Loss of Met Activating Activity in NK1 Mutants but Not HGF Mutants

**Figure 2:**
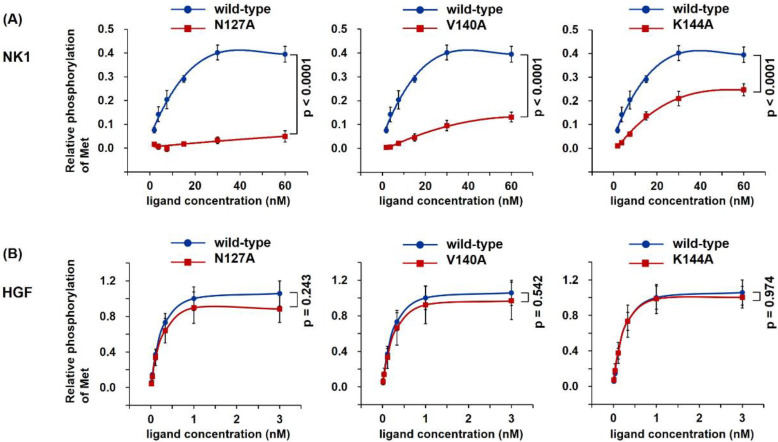
Met receptor activation by wild-type and mutant NK1 (**A**) and HGF (**B**). EHMES-1 cells were treated with recombinant protein for 10 min and Met Tyr1234/1235 phosphorylation was determined by cell-based ELISA. The phosphorylation level induced by 1 nM wild-type HGF was set to 1.0. Data represent mean ± s.e.m [*n* = 3, independent experiments, two-way analysis of variance (ANOVA)].

To determine the biological ability to activate Met, recombinant proteins were tested by Met tyrosine phosphorylation assays in human mesothelioma cells (Figure 2). Met Y1234/1235 phosphorylation, a key Met activation event upon HGF stimulation, was evaluated by cell-based ELISA [8]. The addition of wild-type NK1 induced concentration-dependent Met activation (Figure 2A); The highest level of Met activation was achieved by 30 nM wild-type NK1. In comparison, mutant NK1 showed a decreased ability to activate Met. K144-NK1 showed appreciable Met activation while V140-NK1 showed slight activation, but N127-NK1 hardly activated Met at all. Upon comparing the activities of wild-type and mutant NK1 at 30 nM, N127-NK1, V140-NK1, and K144-NK1 showed 8.3%, 23.8%, and 52.2% activity, respectively, compared with wild-type NK1 (Figure 2A). Met activation by wild-type HGF was 2.5-fold higher than that seen with 30 nM wild-type NK1 (Figure 2B). In contrast to the mutant NK1 proteins that showed decreased activity for Met activation, all mutant HGF proteins exhibited Met activation at a level indistinguishable from that of wild-type HGF. 

The lack of Met activation by NK1 mutants is probably due to the lack of Met dimerization. To analyze Met dimer formation, wild-type and N127A mutant of HGF or NK1 were added to cells in culture, the cells were treated with a chemical cross-linker, and Met was analyzed by immunoprecipitation and Western blotting (Figure S4) [8,27]. Wild-type NK1 facilitated Met dimer formation, whereas N127 mutant NK1 did not. Both N127 mutant and wild-type HGF strongly facilitated Met dimer formation in an indistinguishable manner. These results suggested that the dimer formation of NK1 is required for Met activation, whereas Met activation by HGF does not depend on a specific structure created upon NK1 dimer formation.

### 2.3. Distinct Activation of the Met-Related Signaling Pathway by NK1 and HGF Mutants

Previous studies showed that HGF, NK1, and NK2 have different biological activities. HGF and NK1, but not NK2, induce epithelial tubulogenesis [28]. NK2 enhances the migration of cells and antagonizes the mitogenic response induced by HGF or NK1 [29,30,31]. A synthetic Met agonist composed of cyclic peptide weakly activated Met but enhanced cell motility at levels comparable to that of HGF [32]. These results indicate that Met activation induced by different ligands with different structures may lead to distinguishable Met-dependent biological activities, perhaps because of the different structures in Met for activation. Therefore, to confirm the significance of the dimer interface by NK1 in HGF-dependent Met activation, we assessed the effect of the wild-type and mutant NK1 and HGF proteins on Met-mediated intracellular signaling and biological responses.

**Figure 3:**
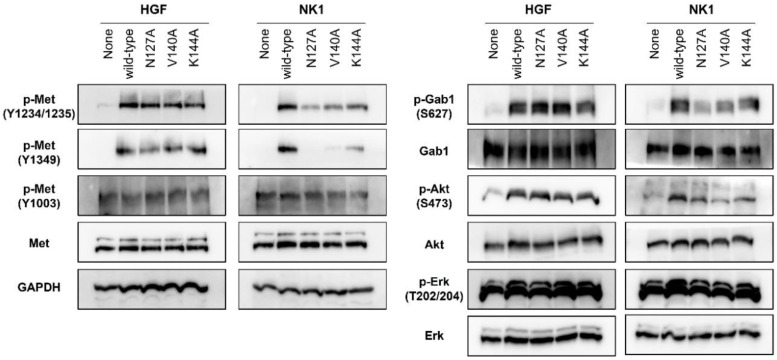
Phosphorylation profiles of Met signaling pathway by wild-type and mutant NK1 or HGF proteins. Serum-starved EHMES-1 cells were stimulated with the recombinant proteins and 5% fetal bovine serum for 10 min. Phosphorylated individual residues of Met receptors, Gab1, Akt, and Erk were detected by Western blotting. GAPDH (glyceraldehyde-3-phosphate dehydrogenase) was monitored to ensure equal loading. This experiment was performed once.

HGF induced the phosphorylation of Y1234 and Y1235 in the kinase domain, Y1349 and Y1356 in the multifunctional docking site in the C-terminal, and Y1003 in the juxtamembrane domain [1,2,3,33,34]. Consistent with this, wild-type HGF induced Y1234/1235 and Y1349 phosphorylation (Figure 3). N127A, V140A, and K144A mutant HGF also induced Y1234/1235 and Y1349 phosphorylation at levels similar to or slightly weaker than those of wild-type HGF. Y1003 phosphorylation was scarcely changed after stimulation by wild-type and mutant HGF. Both wild-type and mutant HGF enhanced the phosphorylation of Gab-1 (S627), Akt (S473), and Erk (T202/204) to similar levels. Wild-type NK1 increased the phosphorylation of Y1234/1235 and Y1349 in Met, Gab-1, Akt, and Erk; however, N127A, V140A, and K144A mutant NK1 showed decreased activity to promote the phosphorylation of Met Y1234/1235 and Y1349, Gab-1, Akt, and Erk. N127A mutant NK1 showed the most decreased activity to induce these phosphorylations. Thus, the levels of Met Y1234/1235 and Y1349, Gab-1, Akt, and Erk phosphorylation by wild-type and mutant HGF or NK1 were consistent with the dose-dependent Met phosphorylation (Figure 2).

### 2.4. Loss of Biological Activity to Promote Cell Motility in NK1 Mutants but Not HGF Mutants

**Figure 4:**
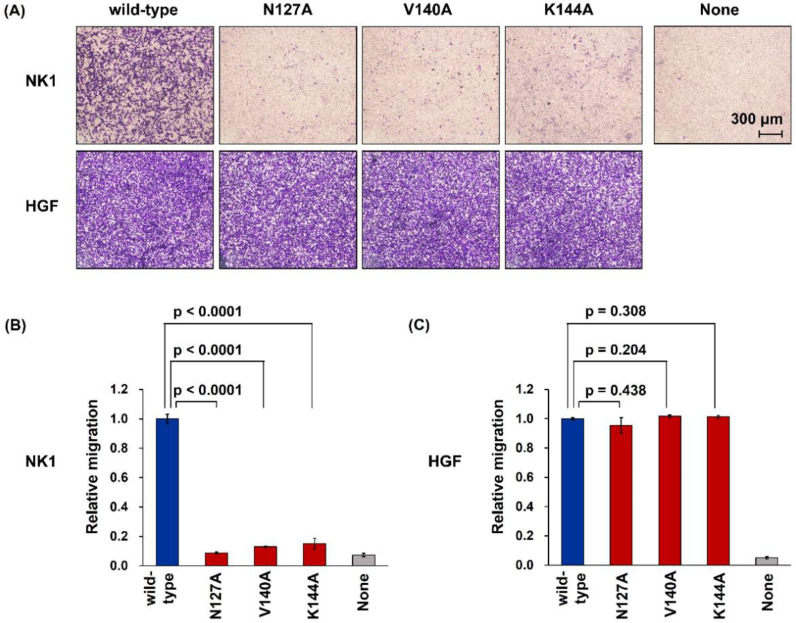
Cell migration induced by wild-type and mutant NK1 (**A**,**B**) and HGF (**A**,**C**). B16F10 cells were cultured in the absence or presence of 35 nM NK1 or 0.2 nM HGF for 20 h. In A, representative images of the migrated cells on the bottom side of the membrane after crystal violet staining are shown. In B and C, quantification of migrated cells is shown. Cell migration levels induced by wild-type NK1 or HGF were set to 1.0. Data are mean ± s.e.m. (*n* = 3, distinct replicates for cell cultures, unpaired two-tailed t-test). This experiment was repeated twice independently with similar results.

To determine the biological activity of mutant NK1 and HGF in regulating cell migration, B16F10 murine melanoma cells seeded in Transwell chambers were incubated with recombinant proteins, and the cells that migrated through the membrane were quantified. Wild-type NK1 strongly enhanced the migration of cells (Figure 4A,B). All NK1 mutants, N127-NK1, V40-NK1, and K144-NK1, showed marginal activity to enhance cell migration or lost such activity altogether; N127-NK1 almost completely lost the ability to enhance cell motility, while V140-NK1 and K144-NK1 showed marginal activity. Wild-type HGF remarkably enhanced the migration of the cells (Figure 4A,C). In contrast to the results with mutant NK1 proteins, all mutant HGF proteins, N127-HGF, V140-HGF, and K144-HGF, strongly promoted cell motility at a level similar to that of wild-type HGF.

**Figure 5:**
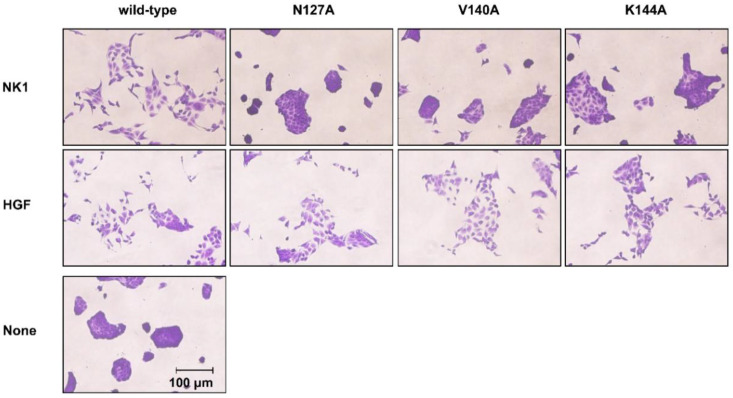
Cell scattering induced by wild-type and mutant NK1 and HGF. MDCK cells were cultured with or without 35 nM wild-type or mutant NK1 or 2 nM HGF for 20 h. The cells were fixed, stained, and then photographed. Representative images of the scattered cells are shown. This experiment was performed once. Bar, 100 μm.

The biological activity of NK1 and HGF to promote cell scattering was determined using MDCK renal tubular cells (Figure 5). The cells were cultured in a 96-well plate with or without wild-type and mutant NK1 and HGF for 20 h. Wild-type NK1 efficiently caused cell scattering. However, N127A and V140A mutant NK1 proteins largely lost their cell-scattering activity. K144A mutant NK1 showed marginal activity to induce cell scattering. By contrast, N127A, V140A, and K144A mutant HGF proteins showed cell-scattering activity comparable to that of wild-type HGF. These findings showed that the mutations in amino acids related to NK1 dimer formation cause loss of NK1-mediated cell migration. However, this was not the case with HGF, even in the presence of mutations of amino acids related to NK1 dimer formation.

**Figure 6:**
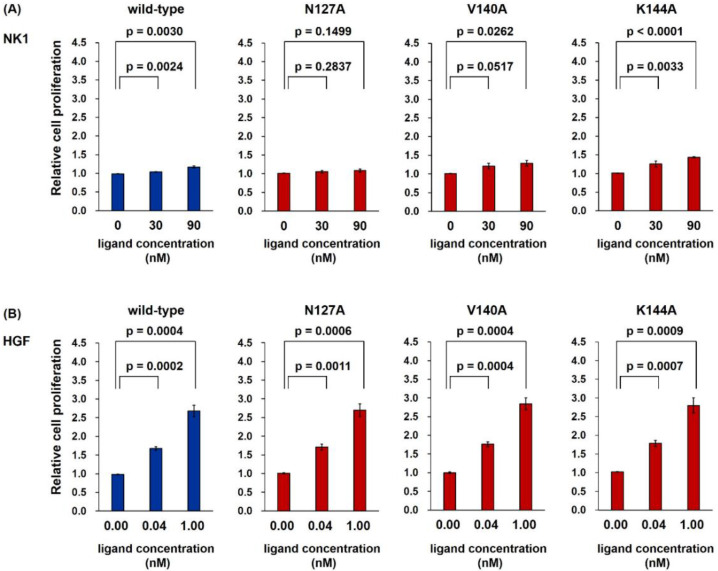
Promotion of cell proliferation by wild-type and mutant NK1 (**A**) or HGF (**B**). Mv1Lu cells were cultured for 72 h, and the value obtained without NK1 or HGF was regarded as 1.0. Data represent mean ± s.e.m. (*n* = 3, unpaired two-tailed t-test). The experiment was independently repeated twice, and similar results were obtained.

We next performed a cell proliferation assay using Mv1Lu lung epithelial cells (Figure 6). The cells were cultured in the presence or absence of wild-type and mutant NK1 or HGF for 72 h. Wild-type HGF and all mutant HGF proteins promoted cell proliferation, and the activity was indistinguishable between wild-type HGF and mutant HGF. In contrast, both wild-type NK1 and NK1 mutants showed marginal or almost no activity to promote cell proliferation. Thus, changes in the activity of mutant NK1 proteins by the replacement of an amino acid involved in NK1 dimer formation could not be appreciable in the cell proliferation assay. Previous studies also noted that HGF has a much higher activity to promote cell proliferation than NK1 [19,35].

### 2.5. Competitive Action of NK1 Mutant on Cell Scattering

**Figure 7:**
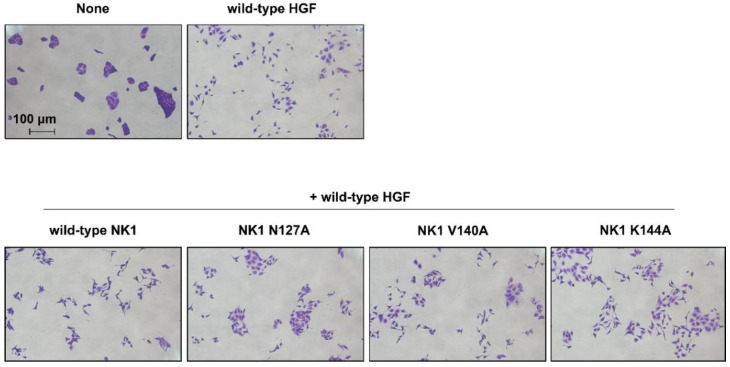
Effects of wild-type and mutant NK1 on HGF-induced cell scattering. MDCK cells were cultured with or without 0.1 nM HGF in the absence or presence of 250 nM wild-type or mutant NK1 for 20 h. The experiment was independently repeated twice, and similar results were obtained.

Considering the competitive action of HGF variants capable of binding to Met but not capable of activating it, we tested whether the NK1 mutants could inhibit the HGF-mediated biological activity using a cell-scattering assay (Figure 7). HGF induced clear cell scattering, whereas N127A-NK1 mutant significantly inhibited the HGF-induced cell scattering, albeit not completely. V140A-NK1 and K144A-NK1 weakly inhibited HGF-induced cell scattering. Because wild-type NK1 has its own activity to enhance cell scattering, wild-type NK1 exerts no inhibitory action on HGF-induced cell scattering. Mutant NK1 proteins have appreciable inhibitory effects on HGF-induced cell scattering, while N127A-NK1 mutant with markedly decreased activity in Met activation exhibits competitive inhibitory activity on HGF-induced cell scattering. 

## 3. Discussion

HGF is biosynthesized and secreted as single-chain HGF (scHGF), and proteolytic cleavage between R494 and V495 results in its conversion to two-chain HGF (tcHGF) (HGF refers to tcHGF unless otherwise noted) [12,13]. Both scHGF and tcHGF bind to Met, but only tcHGF activates it, which has been puzzling regarding the structural basis for Met’s activation by HGF [36,37,38]. After the cleavage of scHGF, the newly formed N-terminal region of the β-subunit is inserted into a cavity in the SP domain that allosterically changes the conformation of the SP domain and enables the SP domain to bind to Met and tcHGF to activate Met [39,40,41]. In addition to this local conformational change, direct observation of scHGF and tcHGF by high-speed atomic force microscopy indicated that the inter-domain connection at the K4–SP domain is organized differently between scHGF and tcHGF, while other inter-domain connections are flexible [10]. This is also supported by SAXS analysis of scHGF and tcHGF [22]. Thus, the cleavage-dependent local conformational change in the SP domain and the rearrangement of the inter-domain connection at the K4–SP domain are associated with the ability of the SP domain to bind Met and for tcHGF to activate it. In contrast, there is no clear difference in the structure and Met binding ability in the NK4 regions between scHGF and tcHGF. In addition, despite mutations in the cleavage-independent constitutive Met binding site in the K1 domain of HGF, the ability of mutant HGF to activate Met was maintained, even though its binding to Met was lost [23]. These results indicated that Met binding in the K1 domain is not required for Met activation, while the NK1 domain is required for Met activation because tcHGF with NK1 deletion cannot activate Met [23,38,42]. Thus, the function of NK1 in Met activation by HGF remains to be further addressed.

The assumption that NK1:Met in a 2:2 complex in which the head-to-tail NK1 dimer provides the core structure is reasonable and the most likely structural basis for Met activation by NK1 because (1) NK1 provides the Met binding interface in HGF [15,16,23], (2) the NK1 dimer structure has been elucidated by crystallographic analysis [24], and (3) the replacement of amino acids responsible for NK1 dimer formation impairs NK1-dependent Met activation, as shown in this study. However, our results showed that replacement of the same amino acids responsible for NK1 dimer formation in HGF did not affect Met activation by HGF, suggesting that the NK1 region does not serve as a dimer interface in HGF-dependent Met activation. How the Met dimer is induced by HGF remains unclear. Independent approaches by electron microscopy, SAXS, and size-exclusion chromatography analysis indicated a 1:1 complex between HGF and the Met ectodomain [22,23], while SAXS analysis with HGF and Sema-PSI domains, but not the whole Met ectodomain, suggested the 2:2 complex as the most likely model [22]. Live-cell imaging experiments also suggested the formation of a 2:2 complex on the cell surface [43]. Recently, a complex structure between HGF and Met ectodomain was shown by cryo-EM analysis [21]. In this model, one HGF molecule associates with two Met molecules utilizing two individual binding interfaces in the SP domain and K1 domain of HGF; in this way, HGF and Met form a 1:2 complex. This study also provided a 2:2 model for the NK1–Met complex as determined by cryo-EM analysis, wherein NK1 forms a head-to-tail dimer and recruits two Met molecules symmetrically to both sides [21]. This complexity explains why an understanding of the structural basis of Met activation by HGF has remained elusive, particularly in living cells. Our results indicate that NK1 dimer is indispensable in NK1-induced Met activation, whereas NK1 dimer structure is dispensable for HGF-induced Met activation. Our results do not contradict the latest structures obtained by cryo-EM [21].

Over the last few years, different types of Met-agonist/HGF-mimetic molecules have been generated, including monoclonal antibodies, macrocyclic peptides, and DNA aptamers [8,9,32,44,45]. Met binders by macrocyclic peptides and DNA aptamers are engineered to be bivalent. Although structures for Met activation achieved by these non-native HGF mimetics have not been defined, the linker length between two Met-binding moieties has some flexibility [8,9,32]. The structure and mechanism for Met activation may involve certain flexibility in response to different ligand structures rather than a single structure and mechanism. Ligand-independent Met activation caused by Met gene amplification has been noted in cancer cells, but the mechanism behind this has remained unknown.

In summary, the replacement of amino acids involved in the head-to-tail dimer formation in NK1 impaired NK1-dependent Met activation, but replacement of the same amino acids in HGF did not affect HGF-dependent Met activation. Our results indicate that the structural basis for inducing NK1-dependent Met dimer formation and activation is distinct from, or at least distinguishable from, the structural basis for inducing HGF-dependent Met activation. Met activation is expected to become a mechanism for regeneration-based therapeutics, while Met inhibition, particularly selective to Met activation-addicted cells, is anticipated to become a method for cancer therapeutics. Further elucidation of the structural basis for ligand-dependent/-independent Met activation may facilitate the discovery and molecular design of both agonists and inhibitors of Met.

## 4. Materials and Methods

### 4.1. DNA Construction

For the full-length HGF, human HGF cDNA (NM_001010932.2) was used. The numbering of amino acid residues was based on variant 3, which lacks five amino acids in the K1 domain, compared with variant 1. NK1 cDNA (residues Met1 to Glu210) was prepared using the full-length HGF. 

HGF and NK1 cDNAs with or without point mutations in the K1 domain (V140A, K144A) and the linker between the N and K1 domains (N127A) were cloned into the pEHX1.1 plasmid (Toyobo, Osaka, Japan). Mutations in NK1 cDNA were introduced by site-directed mutagenesis using the following primer sets: “Asn127Ala”, “Val140Ala”, and “Lys144Ala”. The sequences of the primer sets are listed in Table S1 (Supplementary Materials). For mutations in HGF cDNA, the cDNAs for NK1 mutants were amplified using the primer set “NK1 fragment for HGF” (Table S1). The amplified fragments were used for plasmid construction of mutant HGF cDNA by in-fusion cloning (Takara Bio, Kusatsu, Japan) integrating the two DNA fragments amplified from the pEHX1.1 plasmid containing wild-type HGF cDNA, using the following primer sets: “in-fusion cloning-1” and “in-fusion cloning-2” (Table S1).

### 4.2. Recombinant Protein Preparation and Analysis

The expression constructs were transfected into Expi293F cells (ThermoFisher Scientific, Waltham, MA, USA) using the ExpiFectamine 293 Transfection Kit (ThermoFisher Scientific). Recombinant proteins were purified with HiTrap Heparin HP (1 mL; GE Healthcare, Chicago, IL, USA), using a concentration gradient of NaCl in buffer composed of 50 mM Tris-HCl (pH 7.5) and 0.01% Tween80. For wild-type and mutant HGFs, fractions containing HGF proteins were collected and incubated with 5% fetal bovine serum (FBS) at 37 °C overnight to prepare two-chain HGF. Fractions containing NK1 or HGF were subjected to sulfopropyl (SP) Sepharose Fast Flow (1 mL; GE Healthcare) cation exchange chromatography. Proteins were eluted using a concentration gradient of NaCl in a buffer composed of 50 mM Tris-HCl (pH 7.5) and 0.01% Tween80. Fractions were analyzed by sodium dodecyl sulfate-polyacrylamide gel electrophoresis (SDS-PAGE) followed by protein staining with Coomassie Brilliant Blue (CBB) or Western blot analysis. Protein concentration was measured by the BCA assay (ThermoFisher Scientific).

Proteins were subjected to SDS-PAGE, followed by protein staining with CBB (Rapid Stain CBB kit; Nacalai Tesque, Kyoto, Japan). For Western blotting, after SDS-PAGE, the proteins were electroblotted onto a polyvinylidene difluoride membrane. The membrane was blocked in Tris-buffered saline (TBS) containing 0.05% Tween 20 (TBST) with 3% (*w*/*v*) bovine serum albumin (BSA) for 1 h at room temperature and then treated with rabbit polyclonal anti-human HGF IgG in TBST containing 3% goat serum overnight at 4 °C. After washing with TBST, the membrane was incubated with horseradish peroxidase (HRP)-conjugated anti-rabbit IgG (Dako, Carpinteria, CA, USA) for 1 h at room temperature. Chemiluminescence was visualized using an ImmunoStar LD (Fujifilm Wako Pure Chemicals, Osaka, Japan) and detected using Fusion Solo S (Vilber Lourmat, Marne la Vallée, France).

Concentrations of wild-type and mutant NK1 and HGF were determined by ELISA. Wells in a 96-well plate (Greiner Bio-One, Frickenhausen, Germany) were coated with 10 μg /mL rabbit polyclonal anti-human HGF IgG overnight at 4 °C. The plates were washed with TBST, blocked with 3% (*w*/*v*) BSA in TBST, and then incubated with test samples for 1 h at room temperature. After washing with TBST, wells were incubated with 2 μg /mL biotinylated rabbit polyclonal anti-human HGF IgG for 1 h. After washing with TBST, wells were incubated with HRP-conjugated streptavidin (ThermoFisher Scientific) for 30 min. The enzyme reaction was performed using H_2_O_2_ and o-phenylenediamine (Fujifilm Wako Pure Chemicals) as substrates. The reaction was analyzed by measuring absorbance at 490 nm. Purified wild-type NK1 and HGF were used as the standard.

### 4.3. Cell-Based Met Phosphorylation Assay

EHMES-1 human mesothelioma cells were supplied by Dr. Hamada (Ehime University, Matsuyama, Japan) and cultured in RPMI-1640 medium supplemented with 10% FBS at 37°C and 5% CO_2_. Cells were seeded at 8.0 × 10^3^ cells per well in a 96-well black mClear-plate (Greiner Bio-One, Frickenhausen, Germany) and cultured overnight. Cells were stimulated with each recombinant protein in 50 mM Tris-HCl (pH 7.5), 0.01% Tween80, and 150 mM NaCl supplemented with 5% FBS for 10 min. After washing with ice-cold phosphate-buffered saline (PBS), cells were fixed with 4% paraformaldehyde in PBS for 30 min. After three washes with PBS, cells were blocked with 5% goat serum and 0.02% Triton X-100 in PBS for 30 min, and then incubated with anti-phospho-Met (Tyr1234/1235) XP rabbit monoclonal antibody (Cell Signaling Technologies, Danvers, MA, USA) diluted at 1:1000 in PBS containing 5% goat serum and 0.02% Triton X-100 for 2 h at room temperature. Cells were washed three times with PBS and incubated with HRP-conjugated anti-rabbit IgG (Dako) in PBS containing 1% goat serum for 1 h, followed by three washes with PBS. Chemiluminescence was developed with the ImmunoStar LD reagent (Fujifilm Wako Pure Chemicals) and measured by an ARVO MX plate reader (Perkin Elmer, Norwalk, CT, USA). Relative Met phosphorylation level was calculated as follows: (chemiluminescence unit of sample—chemiluminescence unit of PBS control)/(chemiluminescence unit of 1.0 nM HGF—chemiluminescence unit of PBS control).

### 4.4. Western Blot Analysis

EHMES-1 cells were seeded at 0.67 × 10^5^ cells per well in a 12-well plate and cultured overnight. Cells were washed with PBS and serum-starved in a culture medium without FBS for 6 h. The cells were stimulated in RPMI-1640 supplemented with 5% FBS for 10 min. After washing with ice-cold PBS, the cells were lysed with 100 μL of RIPA lysis buffer containing 1× protease inhibitor cocktail (Nacalai Tesque, Kyoto, Japan), 1 mM PMSF, and 10 mM NaF. The lysates were subjected to SDS-PAGE and Western blotting. Anti-pMet (Y1234/1235; #3077S), -pMet (Y1349; #3133S), -pMet (Y1003; #3135), -Met (#8198S), -pGab1 (Y627; #3233S), -Gab (#3232S), -pAKT (S473; #4060S), -AKT (#9272S), -pErk (T202/Y204; #4377S), -Erk (#4695S), and GAPDH (#2118S) antibodies were purchased from Cell Signaling Technologies). The respective primary antibodies and HRP-conjugated secondary antibody (Dako) were diluted in Can Get Signal Solutions 1 and 2 (at 1:2000 dilution; Toyobo, Osaka, Japan), respectively. Chemiluminescence was visualized by ImmunoStar LD (Fujifilm Wako Pure Chemical) and detected using Fusion Solo S (Vilber Lourmat, Marne la Vallée, France). 

### 4.5. Met Receptor Dimerization in Live Cells

EHMES-1 cells seeded in 60-mm culture plates were cultured until about 90% confluency. Cells were washed twice with ice-cold RPMI-1640 supplemented with 10% FBS and incubated with the test proteins for 1 h at 4 °C. Cells were washed with ice-cold PBS three times and treated with 1 mM bis (sulfosuccinimidyl) suberate (BS3, non-cell-permeable cross-linker; ThermoFisher Scientific) in PBS for 1 h at 4 °C. Non-reactive BS3 was quenched with 50 mM Tris (pH 8.0) and 150 mM NaCl for 15 min at 4 °C. Cells were washed with ice-cold PBS three times and solubilized in lysis buffer (40 mM Tris-HCl (pH 8.0), 1% Triton X-100, 1% NP-40, 10% glycerol, 0.15 M NaCl, 2 mM EDTA, 1 mM PMSF, 1 × Complete protease inhibitor cocktail). Cell lysates were passed through a 27-G needle five times and centrifuged for 15 min at 15,000× *g*. The obtained supernatants were incubated with 1 μg  of anti-MET antibody (sc-514148; Santa Cruz, Dallas, TX, USA) for 4 h at 4 °C and incubated with 25 μL of protein G-coupled magnetic beads (Dynabeads Protein G; Invitrogen, Waltham, MA, USA) for 12 h in a rotating apparatus. The beads were washed three times with lysis buffer and treated with SDS-PAGE sample buffer under reducing conditions. The samples were subjected to SDS-PAGE using a 7.5% gradient gel and Western blotting using an anti-MET antibody (#8198S; Cell Signaling Technologies), followed by HRP-conjugated anti-rabbit IgG (Dako). ImmunoStar LD (Fujifilm Wako Pure Chemical) and Fusion Solo S (Vilber Lourmat) were used to visualize the chemiluminescent reaction.

### 4.6. Migration Assay

B16F10 murine melanoma cells were obtained from the ATCC and cultured in RPMI-1640 medium supplemented with 10% FBS. Cells were seeded at 2.0 × 10^5^ into an insert well (6.5 mm diameter Transwell with 8 μm pores; Corning, Corning, NY, USA) in 200 μL of RPMI-1640 medium supplemented with 1% FBS. In the bottom chamber, 600 μL of RPMI-1640 medium with 1% FBS with or without test proteins was added. Cells were cultured for 20 h and fixed with 4% paraformaldehyde in PBS for 30 min. Cells attached to the bottom side of the membranes were stained with 0.4% crystal violet in 20% methanol and extracted with 200 μL of Extraction Solution (Cell Biolabs, San Diego, CA, USA). The absorbance at 560 nm was measured with an ARVO MX (PerkinElmer, Akron, OH, USA).

### 4.7. Cell-scattering Assay

Madin–Darby canine kidney (MDCK) epithelial cells, which were provided by Dr. Montesano (University of Geneva, Geneva, Switzerland), were seeded at 1000 cells per well in a 96-well plate and cultured in Dulbecco’s Modified Eagle Medium (DMEM) supplemented with 10% FBS overnight. Then, the cells were washed with PBS and cultured in DMEM, including 10% FBS with or without 2 nM wild-type and mutant HGF or 35 nM NK1 proteins. After 20 h, the cells were fixed with 4% paraformaldehyde in PBS for 30 min and stained with 0.4% crystal violet in 20% methanol for 10 min. Scatter competitive assay was performed by the same procedure as in the scatter assay, except that cells were treated with wild-type or mutant NK1 for 30 min, and the concentration of wild-type and mutant NK1 was 250 nM (final concentration) before adding wild-type HGF at 0.1 nM (final concentration).

### 4.8. Cell Proliferation Assay

Mv1Lu mink lung epithelial cells obtained from RIKEN BioResource Center (Tsukuba, Japan) were used for the cell proliferation assay [46,47]. Mv1Lu cells were cultured in Minimum Essential Medium supplemented with 10% FBS and 1% non-essential amino acids. Cells were seeded at 5.0 × 10^3^ per well on a 96-well plate in MEM supplemented with 2% FBS and 0.3 ng/mL TGF-β1 (R & D systems, Minneapolis, MN, USA), and the cells were cultured in the absence or presence of test samples for 72 h. The proliferation of the cells was assayed using a Cell Titer96 Aqueous Cell Proliferation Assay kit (Promega, Madison, WI, USA), in accordance with the manufacturer’s instructions.

### 4.9. Statistics

All statistical analyses were performed using GraphPad Prism software (GraphPad Software, San Diego, CA, USA) on mean values calculated from the averages of technical replicates. Statistical significance was calculated by a two-tailed unpaired t-test or two-way ANOVA, with *p* < 0.05 considered statistically significant. The exact *p* values are reported, except when *p* < 0.0001.

## Supplementary Materials

The following are available online at https://www.mdpi.com/article/10.3390/ijms22179240/s1.

## Abbreviations

HGF	hepatocyte growth factor
SAXS	small-angle X-ray scattering
cryo-EM	cryo-electron microscopy
scHGF	single-chain HGF
tcHGF	two-chain HGF
Gab1	GRB2-associated-binding protein 1
Erk	Extracellular signal-regulated kinase
Akt	Protein kinase B
GAPDH	Glyceraldehyde-3-phosphate dehydrogenase
