# Supplementary material for: Dimer Interface in Natural Variant NK1 Is Dispensable for HGF-Dependent Met Receptor Activation
Source: Int J Mol Sci. 2021 Aug 26;22(17):9240. doi: 10.3390/ijms22179240 (PMC8431453; doi:10.3390/ijms22179240)
Supplement: Supplementary file 1 [file ijms-22-09240-s001.zip › ijms-1025504-supplementary.pdf]

Supplementary Materials

Table S1. Primer sets used for the construction of plasmid DNA.

Primer Set	Sequences
**Asn127Ala**	5′-ACTACATTAGAGCATGCATCATTGGT-3′
	5′- ACCAATGATGCATGCTCTAATGTAGT-3′
**Val140Ala**	5′-TACAAGGGAACAGCATCTATCACTAAGA-3′
	5′-TCTTAGTG ATAGATGCTGTTCCCTTGTA-3′
**Lys144Ala**	5′- TCTATCACTGCAAGTGGCATCA-3′
	5′- TGATGCCACTTGCAGTGATAGA-3′
**NK1 fragment for HGF**	5′-ATGTGGGTGACCAAACTCCT-3′
	5′-TTCAACTTCTGAACACTGAGGAAT-3′
**in-fusion cloning-1**	5′-GACAATAACCCTGATAAATGCTTC-3′
	5′-CATTTATCAGGGTTATTGTCTCATG-3′
**in-fusion cloning-2**	5′-TTTGGTCACCCACATGGT-3′
	5′-TGTTCAGAAGTTGAATGCATGA-3′

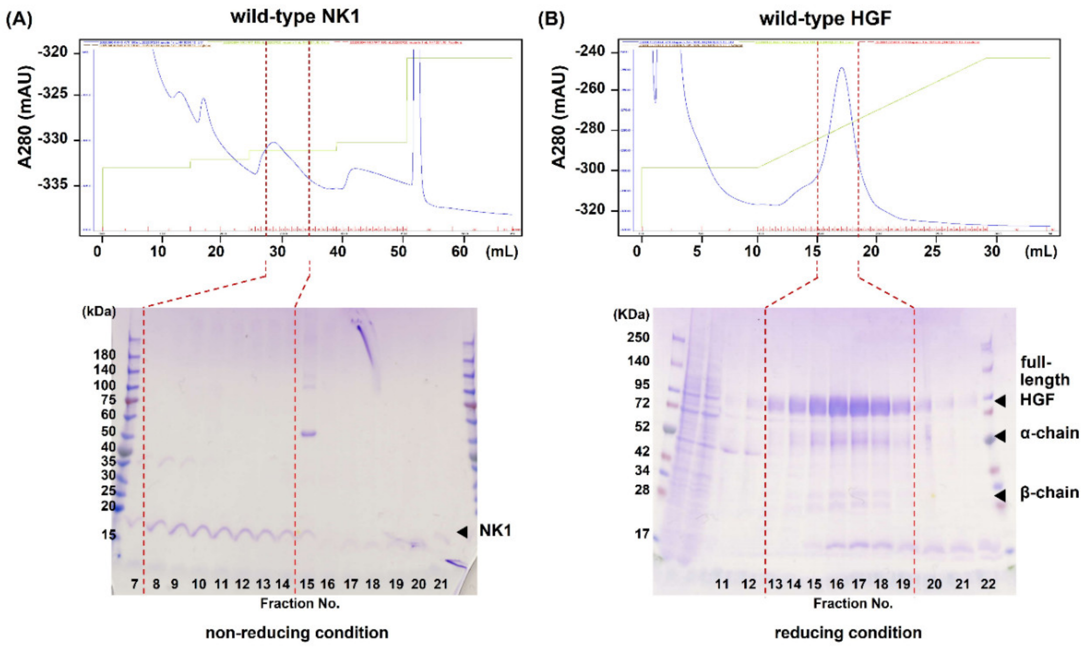

Figure S1. Elution profiles and purification of wild-type NK1 (**A**) and wild-type HGF (**B**) in heparin-affinity chromatography. CBB staining of proteins after SDS-PAGE is shown in the bottom panels. Elution profiles of mutant NK1 and HGF were similar to those of wild-type NK1 and HGF.

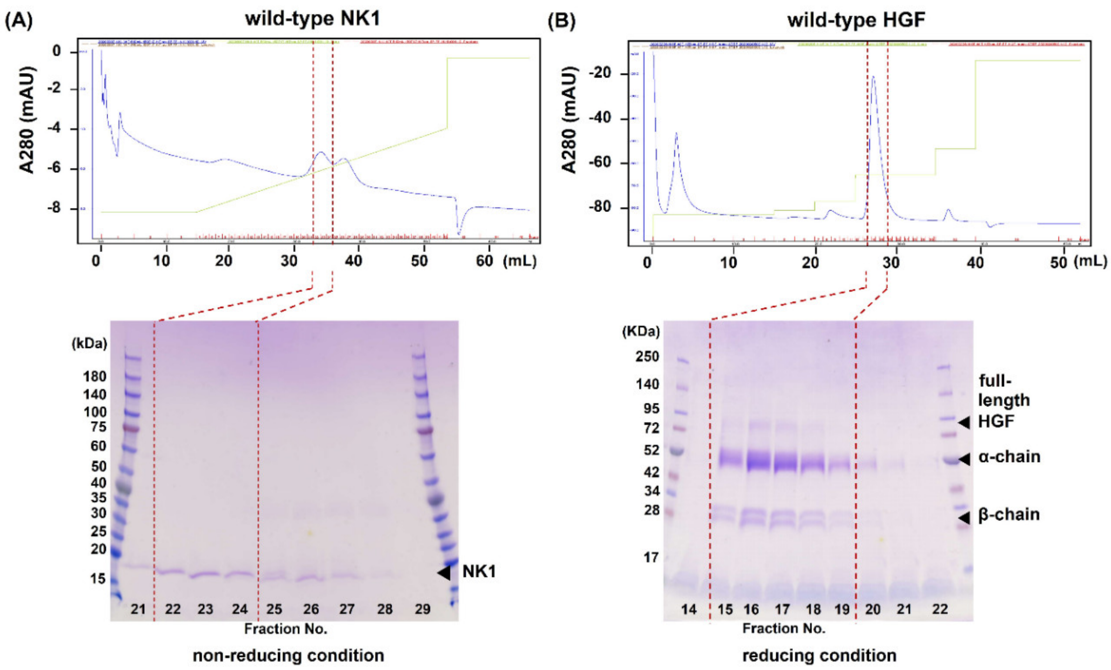

Figure S2. Elution profiles and purification of wild-type NK1 (**A**) and wild-type HGF (**B**) in cation exchange chromatography. CBB staining of proteins after SDS-PAGE is shown in the bottom panels. Elution profiles of mutant NK1 and HGF were similar to those of wild-type NK1 and HGF.

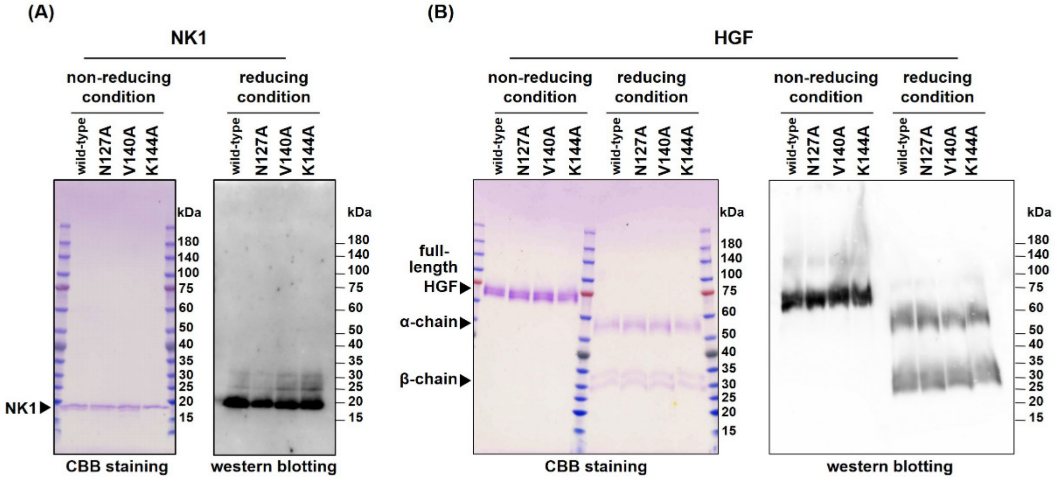

Figure S3. Recombinant NK1 (**A**) and HGF (**B**) confirmed by protein staining and Western blotting. Recombinant proteins were electrophoresed on a 5–20% gradient gel under non-reducing and reducing (by 2-mercaptoethanol) conditions. Proteins were stained with Coomassie Brilliant Blue (CBB) (left) and detected by Western blot analysis (right). Wild-type and mutant HGF show a single band and two bands in non-reducing and reducing conditions, respectively.

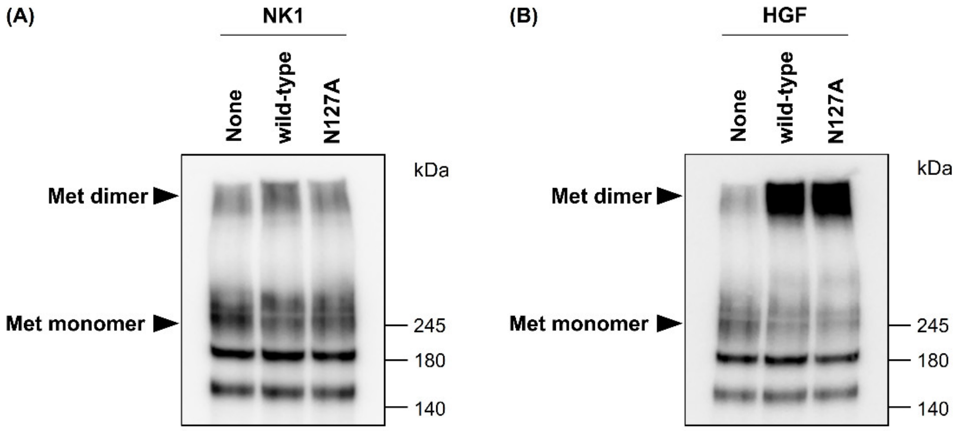

Figure S4. Met dimer formation promoted by NK1 and HGF. EHMES-1 cells were left untreated or treated with 30 nM wild-type and mutant NK1 or 1 nM wild-type and mutant HGF. Met dimer formation was analyzed by chemical cross-linking, immunoprecipitation, SDS-PAGE, and Western blotting.
